# Identification and Fine Mapping of a Stably Expressed QTL for Cold Tolerance at the Booting Stage Using an Interconnected Breeding Population in Rice

**DOI:** 10.1371/journal.pone.0145704

**Published:** 2015-12-29

**Authors:** Yajun Zhu, Kai Chen, Xuefei Mi, Tianxiao Chen, Jauhar Ali, Guoyou Ye, Jianlong Xu, Zhikang Li

**Affiliations:** 1 Institute of Crop Sciences/National Key Facility for Crop Gene Resources and Genetic Improvement, Chinese Academy of Agricultural Sciences, Beijing, 100081, China; 2 Agricultural Genomics Institute, Chinese Academy of Agricultural Sciences, Shenzhen, 518120, China; 3 Shenzhen Institute of Breeding and Innovation, Chinese Academy of Agricultural Sciences, Shenzhen, 518120, China; 4 International Rice Research Institute, DAPO box 7777, Metro Manila, the Philippines; China National Rice Research Institute, CHINA

## Abstract

Cold stress is one of the major abiotic stresses that impede rice production. A interconnected breeding (IB) population consisted of 497 advanced lines developed using HHZ as the recurrent parent and eight diverse elite *indica* lines as the donors were used to identify stably expressed QTLs for CT at the booting stage. A total of 41,754 high-quality SNPs were obtained through re-sequencing of the IB population. Phenotyping was conducted under field conditions in two years and three locations. Association analysis identified six QTLs for CT on the chromosomes 3, 4 and 12. QTL *qCT-3-2* that showed stable CT across years and locations was fine-mapped to an approximately 192.9 kb region. Our results suggested that GWAS applied to an IB population allows better integration of gene discovery and breeding. QTLs can be mapped in high resolution and quickly utilized in breeding.

## Introduction

Cold stress is one of the major adverse environmental factors that limit the growth, productivity, and geographical distribution of crops [1]. Rice (*Oryza sativa* L.) is one of the most important food crops and provides staple foods for about half of the world population. Rice is more sensitive to cold stress than other cereal crops such as wheat (*Triticum aestivum* L.) and barley (*Hordeum vulgare* L.) because of its origin in tropical and subtropical regions [2]. Due to climate fluctuations in recent years, cold stress has been a common problem in rice cultivation worldwide. In China, almost all rice production areas are highly vulnerable to cold injury caused by low temperatures at different developmental stages. The estimated annual lost was 3–5 million tons in China [3]. In high-latitude or high-altitude regions such as Japan and Korea, cold stress caused by low-temperature air or cold irrigation water also occurs frequently and resulted in dramatic grain yield reduction once every several years [4–5]. Thus, rice cultivars with high cold tolerance (CT) are essential for stabilizing the rice production in above-mentioned areas.

Over years several researchers have established many growth-stage specific criteria for the evaluation and selection for CT in rice [2, 6–8]. Evaluation of CT for rice typically takes place during seedling and booting stages [2]. Rice plants are more sensitive to cold stress at the booting stages than at the seedling stage [6–7]. CT at the booting stage is also more important than at the seedling stage, because CT at booting stage is critical for pollen survival, seed set, and grain filling to ensure maximal yield [8].

To implement marker-assisted selection, genetic mapping has been widely used to identify QTLs with large effects and markers tightly linked to these QTLs over the past decades. A wide range of segregating populations derived from bi-parental crosses, including recombinant inbred lines (RILs), doubled haploid lines (DH), F_2_, and BC, have been developed and used for identifying QTL through linkage mapping [9–10]. Linkage mapping using a bi-parental population benefits from high statistical power due to many individuals sharing the identical genotype at arbitrary location, but suffers from low resolution due to the limited number of generations [11]. More than 250 QTLs for CT were identified on all 12 chromosomes using different bi-parental mapping populations and evaluation indexes [12]. Fifty-nine were QTLs for CT at booting stages and individually explained 0.8%–37.8% of the phenotypic variation [12]. Out of the 59 QTLs, only *qCT8* [13], *qCT7* [14] and *qLTB3* [4] were fine mapped and *Ctb1* [15] was cloned. An alternative QTL mapping approach is association mapping. Association mapping is based on natural population or breeding population and has high resolution due to the long recombination histories of natural populations but suffers from low power since most genotypes occur in only a few individuals [11].

To overcome the limitation of single bi-parental population in allelic diversity covered, mapping power and resolution the use of multiple crosses (MC) has recently become popular in QTL mapping. QTL analyses across populations revealed a substantially higher number of QTLs compared to the analyses of single bi-parental populations [16–17]. The joint analyses also allow estimating QTL positions with higher precision [18–19]. There are various designs of the MC population. The most popular one is the NAM population [20–30]. NAM populations have been successfully developed to map QTLs in both allogamous such as maize [20] and sorghum [22] and autogamous such as barley [24].

The development of MC population is time consuming and labor-intensive. It is important to use diverse parental lines of good performance in many traits to allow effective integration of gene discovery and practical breeding. In recent years, our team has adopted a large-scale backcross (BC) breeding strategy to improve multiple abiotic stress tolerance, many interconnected (i.e common recurrent parent) populations were developed and highly selected for one or more agronomic traits [31–34]. Many QTLs controlling complex traits were identified by using the introgression lines (ILs) developed from those BC breeding programs [35–36]. Similar to NAM, it is expected the mapping resolution and power can be significantly increased by the combined use of multiple interconnected breeding (IB) populations. In this paper, an IB population consisting of 497 advanced lines derived from introgression of eight donors into an elite *indica* variety, Huanghuazhan (HHZ), was evaluated for CT at the booting stage to identify stably-expressed QTLs under natural low air temperature condition. A stably-expressed QTL was fine-mapped to a narrow genomic region using NILs identified from the IB population and high density SNP markers generated by re-sequencing. The QTLs identified and the elite lines with CT provided essential information and materials for developing CT rice cultivars by marker-assisted selection (MAS).

## Materials and Methods

### Development of IB population

**Fig 1:**
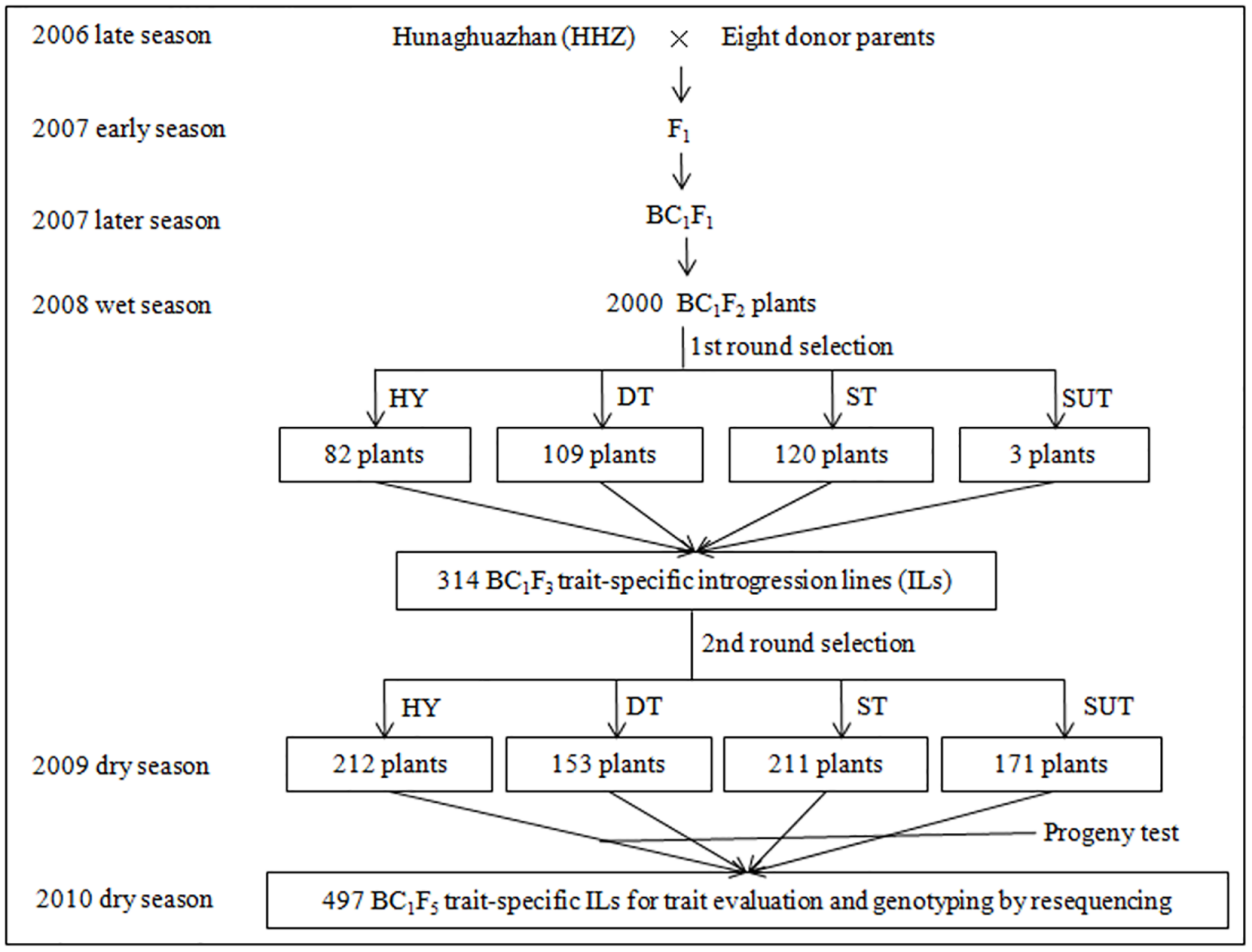
Development of the IB population. Huanghuazhan (HHZ) was used as the recurrent parent and eight diverse varieties as the donor parents. HY, DT, ST and SUT represent high yield under irrigated condition, drought tolerance, salt tolerance and submergence tolerance, respectively.

In 2006, Huanghuazhan (HHZ), a mega *indica* variety in South China with high yielding and quality (http://www.ricedata.cn/variety/), was used as the recurrent parent to cross with eight high yielding donor parents including IR64, PSBRC28, PSBRC66, and IR50 from Philippines, Phalguna from India, OM1723 from Vietnam, and CDR22 and Teqing from China. Eight F_1_s were then backcrossed once with HHZ, later the BC_1_F_1_ selfing seeds were bulk-harvested to produce eight BC_1_F_2_ bulk populations (2000 plants per BC_1_F_2_). Two rounds of screenings for yield and tolerances to various abiotic stresses such as drought, salinity, and submergence were performed at the International Rice Research Institute (IRRI). In all such screenings, surviving plants or plants with higher yield were selected (Fig 1), which showed superior performance over the checks. Total of 314 BC_1_F_3_ trait-specific individuals derived from the 1^st^ round screening were then screened for different target traits again and produced 747 BC_1_F_4_ individuals after the 2^nd^ round of screening. After progeny testing, 497 BC_1_F_5_ introgression lines (ILs) with outstanding performance for at least one target trait were finally selected and used as the IB population for phenotypic evaluation of CT and re-sequencing.

### Phenotyping for CT at the booting stage

Evaluation of CT at the booting stage was performed at three experimental stations of the Yunnan Academy of Agricultural Sciences over two consecutive years, i.e. in Yuxi (23.19°N, 101.16°E, 1,800 m) and Xundian (25.20°N, 102.41°E, 2,500 m) in 2013, and in Xundian and Songming (25.05°N, 102.40°E, 2,136 m) in 2014 under natural low air- temperature condition in Yunnan province, China. The three locations have low temperature air of 15.29–21.42°C during the growth periods starting from booting to milky stages, and are ideal for evaluation of CT for rice [7]. Based on our previous observation of developmental stage of HHZ in above three experimental sites, we arranged a suitable sowing time in each site to ensure HHZ and most ILs hit the low temperature ranging from 17–20°C during periods starting from booting to milky stages. Specifically, seeds were sown on March 14 in Xundian, March 15 in Songming, and March 20 in Yuxi, and then 35-day-old seedlings were transplanted in the field according to a random complete block design with two replicates. The plot size was two rows, 10 plants per row with a spacing of 20 cm between rows and 17 cm between plants. Field management was the same at all locations with three applications of fungicide and insecticide to control disease and brown plant hoppers. Air temperature data were obtained from local meteorological stations as shown in Table 1. The middle eight plants were harvested and three-main panicles per plant were used to measure seed fertility (SF) as the ratio of fertile grains to total grains [7].

**Table 1:** Details of field experiments.

Description	2013		2014	
	Yuxi	Xundian	Xundian	Songming
No. of lines	497	497	497	497
Sowing date	20-Mar	14-Mar	14-Mar	15-Mar
Harvest date	Mid-September	Mid-September	Mid-September	Mid-September
Booting to milky stage	July 2 to August 3	July 4 to August 6	July 7 to August 5	July 11 to August 5
Range of air temperature (°C)	17.05–21.42	16.13–20.40	15.9–20.63	15.29–20.25
Average air temperature (°C)	20.24	18.89	18.84	19.29

### High throughput re-sequencing and SNP identification

Genomic DNA was extracted from the leaf tissues of a single plant from each line using the DNeasy Plant Mini Kit (Qiagen). The library construction and sequencing were performed as described by Huang et al [37]. The 73-mer reads were aligned to the *japonica* Nipponbare reference genome (MSU Release 7.0) using soap version 2.22. The consensus sequences for each sample were generated using SOAPsnp (version1.01) from uniquely aligned reads. These consensus sequences across the whole genome were used to obtain the single-base pair genotypes using SAMtools (version 0.1.18).

The low-quality bases (base-quality *Q* score <20) were removed, and the successfully called sites with conflicting genotypes among different reads were further excluded. Moreover, the overall depth was required to be <15 in each site to avoid mapping to regions with copy-number variation. Next, single-base pair genotypes of the 497 lines were integrated to screen for single nucleotide polymorphism (SNP) markers across the genome. Discrepancies with the reference genome were called as candidate SNPs. Unreliable sites were then filtered according to the following criteria: 1) candidate SNP loci must be more than 10 bp away from each other; and 2) all the singleton SNPs were excluded. Sites passing these criteria were retained and referred as common SNPs (MAF>0.05). Finally, 41,754 high-quality SNPs were selected out from the common SNPs for QTL mapping.

### Statistical analysis

Phenotypic value of each line in each of the testing environments was the average value from two replicates. To avoid the influence of heading date on CT, lines with flowering dates being seven days earlier or later than the population mean were discarded [7]. Phenotypic correlations among four environments were computed using R statistical software [38].

To evaluate the resolution that is expected in our IB population, the linkage disequilibrium (LD) was evaluated by computing the r^2^ values between pairs of SNP markers using TASSEL 5.2 [39]. The window size was set to 1000, so around 1k by 40k pairs of r^2^ was calculated. Since high density markers were used, it is very likely that r^2^ is equal to 1 for two close related markers. Therefore, these values of r^2^ from 40M pairs were filtered for outliers and extreme values. In order to get the relationship between values of r^2^ and the physical distance for the identical marker pair of r^2^, the R-package ggplot2 was used to make a second order polynomial curve fitting for the filtered data.

Association analyses were conducted using the weighted Mixed Linear Model (Weighted MLM) implemented in TASSEL 5.2. The kinship matrix and principal components described previously [40] were used to reduce spurious associations. The threshold to declare a significant association was set at a probability level of 1.0×10^−4^ [41–42]. LD blocks harboring significant SNPs were then defined as the candidate QTLs.

### Fine mapping

Fine mapping was carried out using a physical mapping method as described by Zhu et al. [43]. Near-isogenic lines (NILs) were obtained by first screening the ILs that were covered or partly covered based on the interval of the target QTL using the high-quality SNPs for GWAS. Further, the genetic similarities between these candidate NILs and its recipient parent HHZ were analyzed based on the same SNPs. The lines with genetic similarities > 95% were retained as NILs. Afterwards, saturated density SNPs in the mapping interval of the target QTL, which had MAF>0.03 and miss rate <10%, were selected out from the raw re-sequencing data. Finally, NILs were genotyped using these saturated density SNPs in primary mapping interval of target QTL. For phenotype, two-ANOVA in R [38] was used to reveal the differences between HHZ and NILs.

## Results

### SNP identification

A total of 497 ILs and their nine parents were genotyped. These parental lines were sequenced on average depth of 25X, while the ILs were sequenced in average of 3.16X. SNP calling was conducted based on discrepancies between the consensus sequence and the reference genome. After exclusion of singleton SNPs, a total of 2,256,146 non-redundant SNPs were identified, resulting in an average of 5.4 SNPs per kb, with 86.5% of the SNPs located within 0.2 kb of the nearest SNP. Finally, 41,754 high-quality SNPs with missing rate lower than 10% and minimal MAF higher than 5% were screened out to carry out GWAS.

### Characterization of the IB population

**Fig 2:**
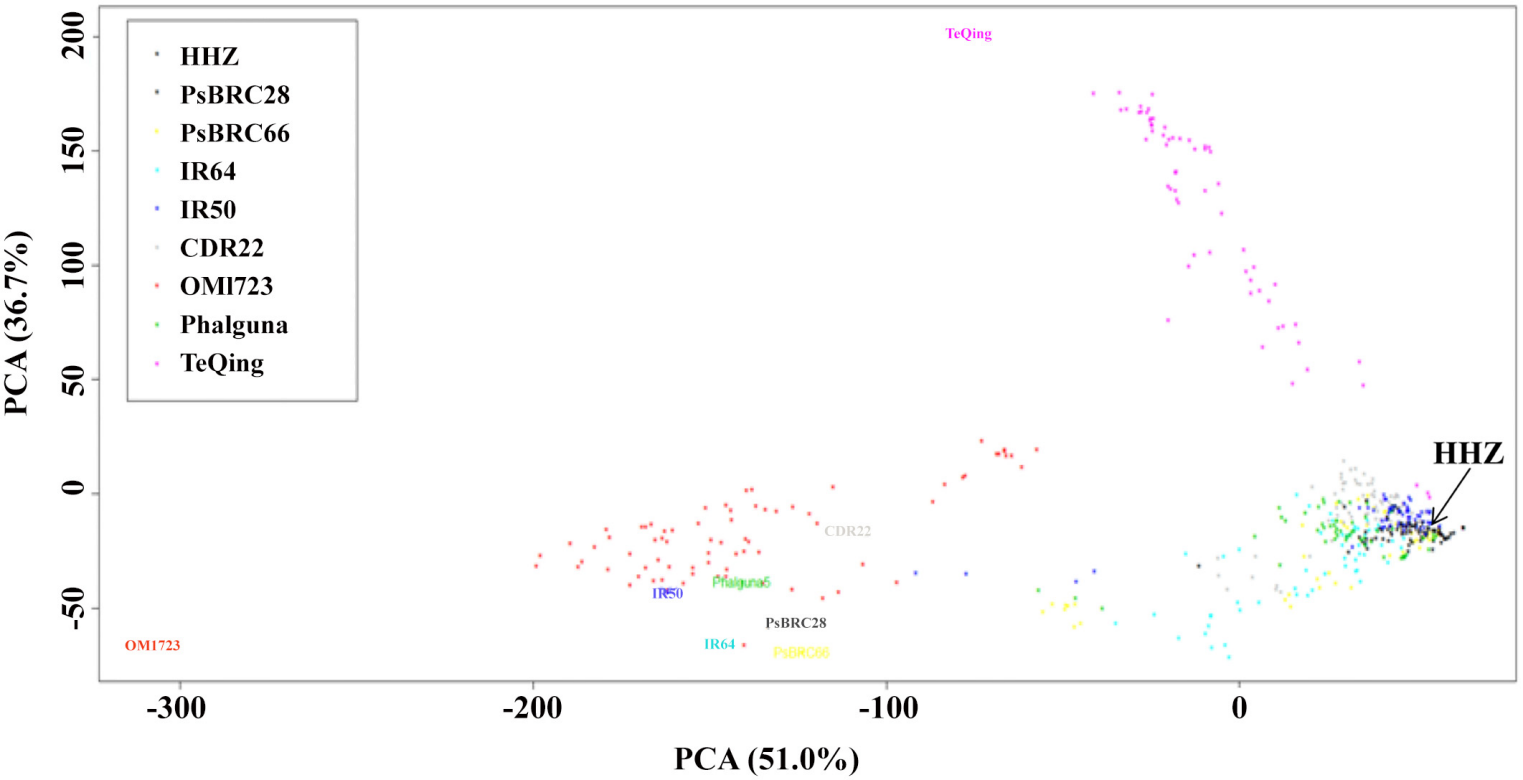
Principal component analysis of the IB population and its parents. The first and the second principal components (PC) are shown as x- and y-axis, respectively. Percentages in brackets denote the variance explained by the respective PC. Donors and IB lines are color-coded based on donor’s geographical origins.

The inheritance of parental segments across the genomes of the 497 lines was characterized through genotyping 41,754 informative SNP markers. Marker saturation was high with an average genetic distance of 9.3 kb. We didn't obtain the LD among parents due to the limited number of samples. The genetic similarities between the nine parents were evaluated. The SNP data revealed that an intermediate genetic similarity between HHZ and donors, ranging from 0.42 to 0.65 (Table 2). Parents could be clearly separated in by principal component analysis (PCA) based on the SNP markers (Fig 2). These results indicated offspring derived from these crosses between HHZ and donors would have abundant allelic variation, which supports high mapping resolution of GWAS. For the IB population, the genetic constitutions of the eight families were displayed fully in S1 Fig. We found the recurrent genome percentage (RGP) showed significant difference among the eight families, with a range of 55.7–89.7% (S1 Table). RPG varied significantly among chromosomes as well, with a range of 68.4–85.5% (S2 Table). The analysis of similarity showed the eight families were different in genetic structure (S2 Fig). The IB population was separated into groups correspondent to the donor parents by PCA (Fig 2). These findings point to the high genetic diversity presented within the IB population and among their parents.

**Table 2:** Cold tolerance of the eight families of the IB population and genomic similarities between HHZ and donors.

Family	No. of lines	Donor	Cold tolerance^1)^	Origin	SF (%)		Genomic Similarity(%)^2)^
					Mean	Range	
H1	53	IR64	S	Philippines	52.17	33.70–68.80	53.7
H2	62	Teqing	S	China	50.50	31.63–74.09	42.2
H3	42	PSBRC66	MS	Philippines	62.99	45.42–76.54	45.4
H4	67	CDR22	S	India	58.77	41.88–70.22	49.3
H5	63	PSBRC28	MS	Philippines	62.97	37.61–74.32	47.2
H6	65	OM1723	S	Vietnam	54.79	30.09–70.14	65.1
H7	53	Phalguna	HS	India	43.02	20.07–74.80	47.0
H8	61	IR50	S	Philippines	51.26	31.25–72.21	53.5

^1)^ S, MS and HS indicate sensitive, moderately sensitive and highly sensitive to cold stress.

^2)^ Genomic similarity with the recurrent parent was estimated by simple matching coefficient based on 41,754 SNPs.

**Fig 3:**
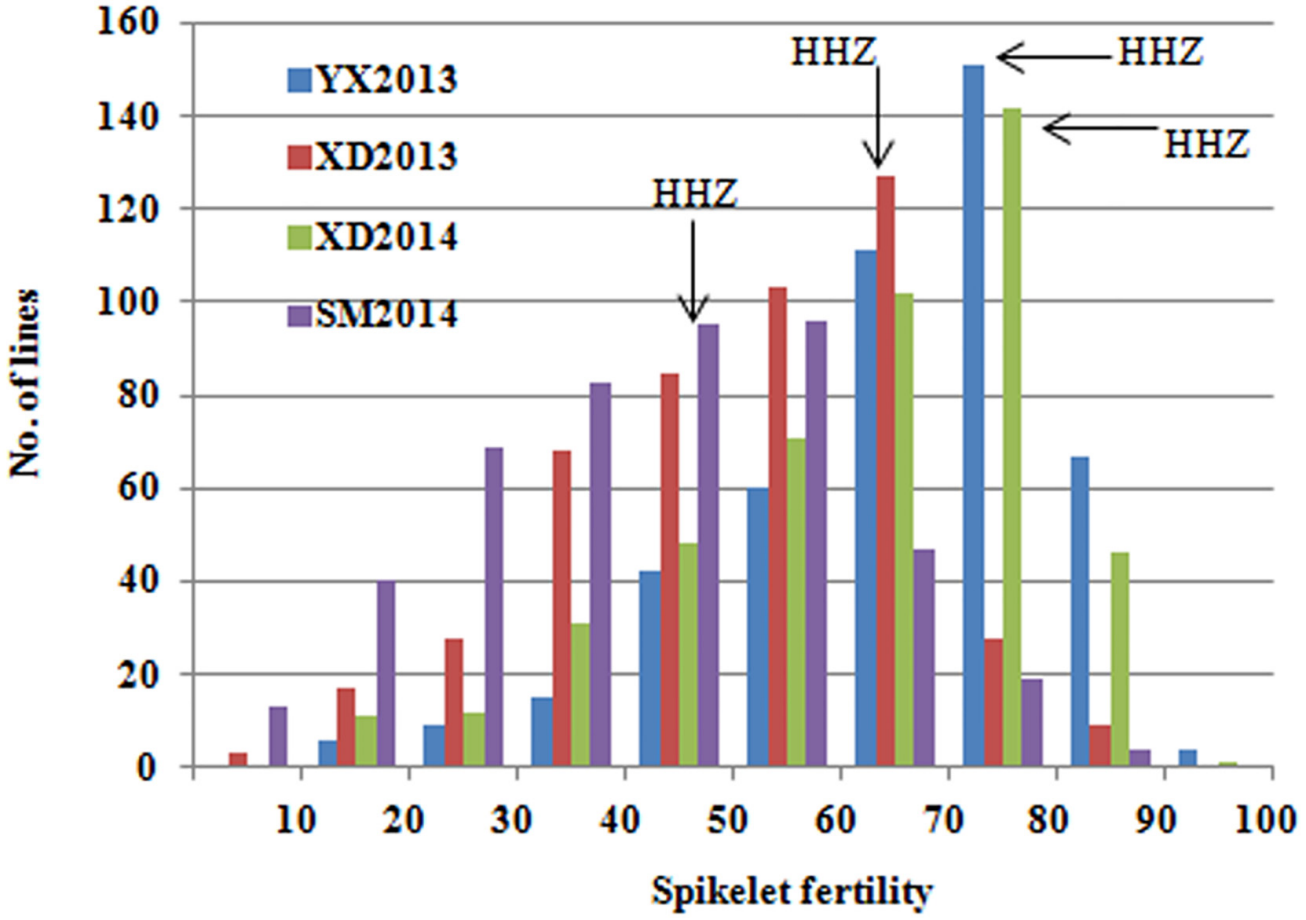
Histogram depicting distribution of spikelet fertilities (SF) of the main panicles IB population and the recurrent parent (HHZ) in four cold stress environments. SF of HHZ is shown with black arrows pointing to the environments.

Diversity was also phenotypically visible (Fig 3). SF ranged from 46.23% to 78.05% with an average of 64.5% (Table 3). After filtering out lines with seven days earlier or later than the population average flowing date, SF of the IB population ranged from 16.70–98.73% (YX2013), 8.96–85.41% (XD2013), 12.4–91.6% (XD2014) and 1.46–79.5% (SM2014), and their corresponding means were 67.1% (YX2013), 51.1% (XD2013), 63.4% (XD2014) and 41.5% (SM2014), respectively (Table 3). Correlation analysis showed that the SF measured in different environments was significantly and positively correlated (S3 Table). The average SF of the IB population was lower than that of the recipient parent (HHZ) in all the four testing environments (Table 3). The eight families of the IB population showed also significant difference in SF. The average SF of family H3, H4 and H5 were higher than those of the others. H7 had the lowest SF (Table 2). These results indicated that the IB population had abundant diversity in CT and some lines may carry QTLs for CT because of their high SF.

**Table 3:** SF of the IB population and the recurrent parent (HHZ) under four test environments.

Environment	SF of IB (%)		SF of HHZ (%)
	Range	Mean	Mean
YX2013	16.7–98.7	67.1±13.8	78.1±4.8
XD2013	9.0–85.4	51.1±15.0	62.9±5.7
XD2014	12.4–91.6	63.4±15.4	70.7±13.6
SM2014	1.5–79.5	41.5±16.4	46.2±4.1
Total	20.1–76.5	55.8±18.2	64.5±14.2

### Association analysis

**Fig 4:**
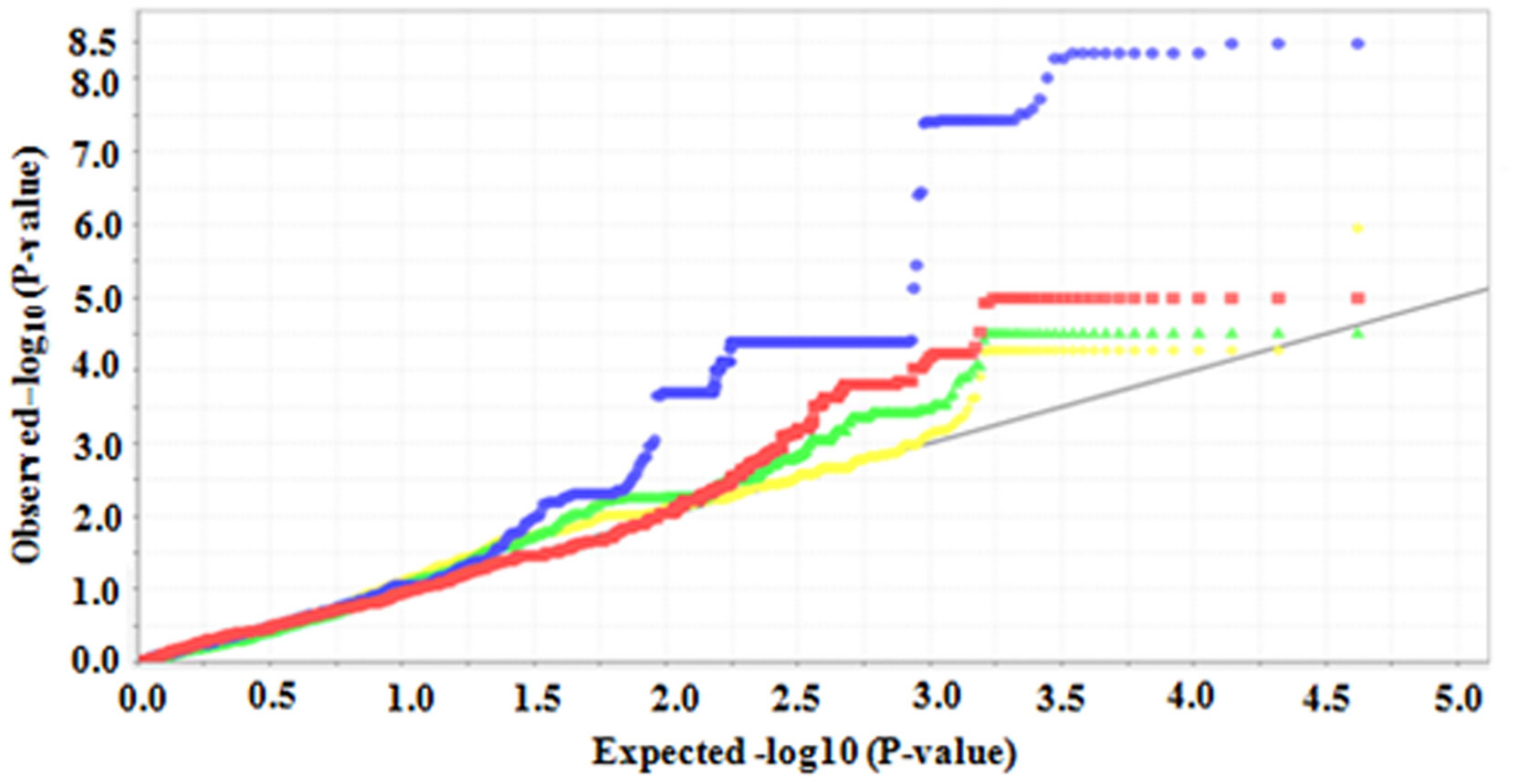
Quantile-quantile plots of cold tolerance tested in four environments. Yellow, green, blue and red are for XD2013, YX2013, XD2014 and SM2014, respectively.

**Fig 5:**
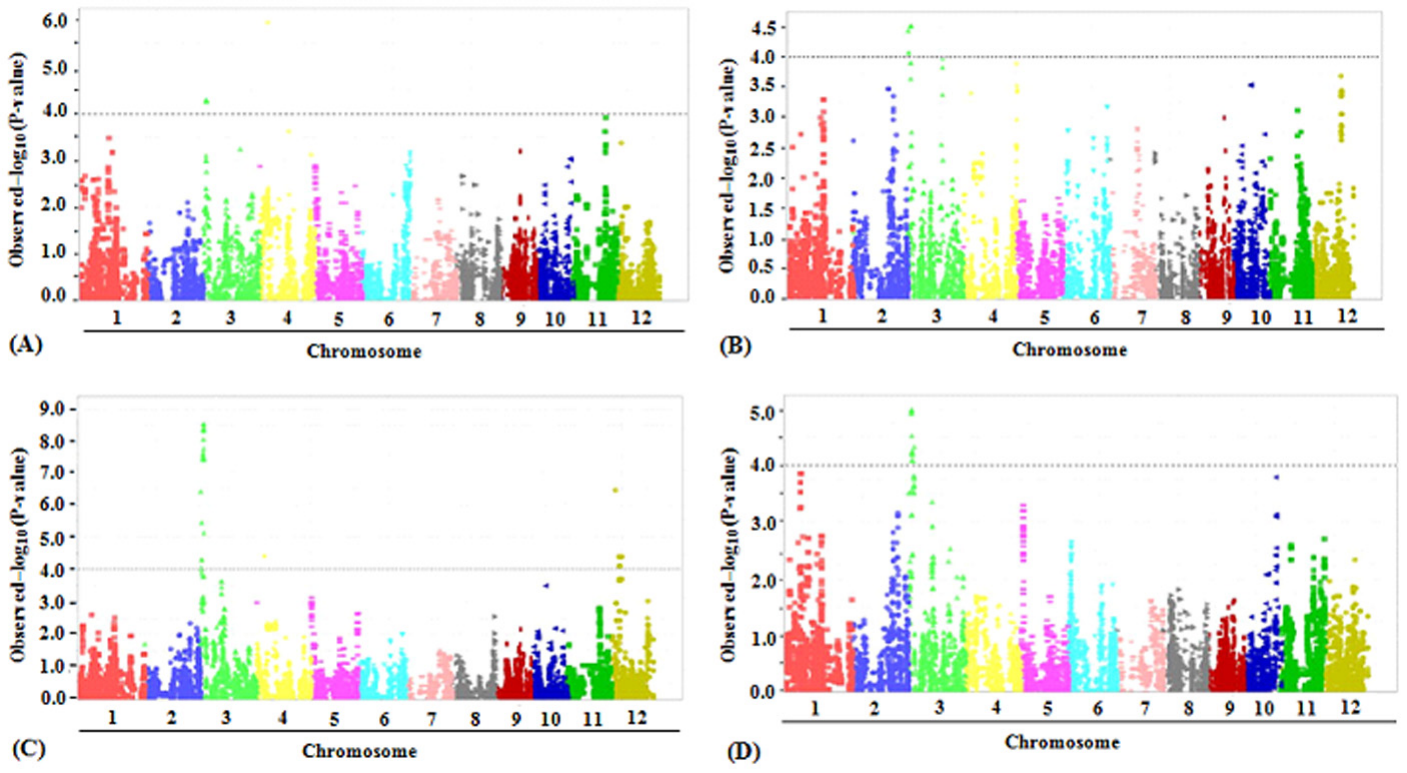
Manhattan plot of mixed linear model analysis for cold tolerance. The letters A, B, C and D indicated XD2013, YX2014, XD2014 and SM2014, respectively.

Total 466 lines were retained from 497 lines for linkage disequilibrium (LD) evaluation and to execute GWAS by removing the lines that were seven days earlier or later than the population mean flowering date. According to r^2^ value, LD value was approximately 400 kb (S3 Fig). Fig 4 gave the Quantile-Quantile plots of for CT in the four testing environments. In total, eight regions were identified as significantly associated with CT (Table 4). The peak markers associated to these regions were anchored on the chromosomes 3, 4 and 12, respectively (Table 4, Fig 5). Taking LD blocks harboring significant SNPs as the candidate loci, six different QTLs were finally revealed (Table 4).

**Table 4:** QTLs identified by GWAS approach under four cold stress environments.

Environment	QTL	Chr.	Peak marker	Physical position (bp)	Association interval (bp)	*P* value	R^2^ (%)
YX2013	*qCT-3-1*	3	Chr03212122	212122	212122–403336	3.62E-05	4.1
**YX2013**	***qCT-3-2***	**3**	**Chr031934364**	**1934364**	**1824682–2150858**	**3.00E-05**	**4.4**
**XD2013**	***qCT-3-2***	**3**	**Chr031935593**	**1935593**	**1824682–2150858**	**5.30E-05**	**4.2**
XD2013	*qCT-4*	4	Chr045026663	5026663	-	1.14E-06	6.7
SM2014	*qCT-3-3*	3	Chr033449080	3449080	3360320–3449080	4.72E-05	4.2
**SM2014**	***qCT-3-2***	**3**	**Chr031934364**	**1934364**	**1770855–2199078**	**1.03E-05**	**5.6**
XD2014	*qCT-3-1*	3	Chr03266205	266205	266205–867091	3.96E-07	6.7
**XD2014**	***qCT-3-2***	**3**	**Chr032108411**	**2108411**	**1770855–2199078**	**3.33E-09**	**9.5**
XD2014	*qCT-4*	4	Chr045026663	5026663	-	3.83E-05	5.1
XD2014	*qCT-12-1*	12	Chr122995458	2995458	-	3.64E-07	8.5
XD2014	*qCT-12-2*	12	Chr125804934	5804934	5759432–6152105	3.64E-07	4.2

Three QTLs, namely *qCT-3-1*, *qCT-3-2* and *qCT-3-3*, were indentified on the short arm of chromosome 3 (Table 4). The *qCT-3-2* was identified in all environments (Table 4). In 2013, it was mapped to a 326.2 kb interval delimited by SNPs Chr031824682 and Chr032150858. In 2014, it was located between SNPs Chr031770855 and Chr032199078. This interval (428.2 kb) covered that of year 2013. In YX2013 and SM2014, *qCT-3-2* had the same peak marker, Chr031934364. The phenotypic variance explained (PVE) by *qCT-3-2* varied from 4.2% to 9.5% (Table 4). Thus, *qCT-3-2* was a stable QTL. The other two QTLs on the chromosome 3 were identified in one and two environments with the PVE being 4.01% and 6.66%, respectively (Table 4). The *qCT-4* associated with SNP Chr045026663 on the chromosome 4 was identified for two times in XD (2013–2014), having the PVE of 6.7% and 5.1%, respectively. On the short arm of chromosome 12, two QTLs were identified in XD2014. The *qCT-12-1* was associated with SNP Chr122995458 with a PVE of 8.5% and *qCT-12-2* was associated with SNP Chr125804934 with a PVE of 4.2%.

### Fine mapping of *qCT-3-2*

**Fig 6:**
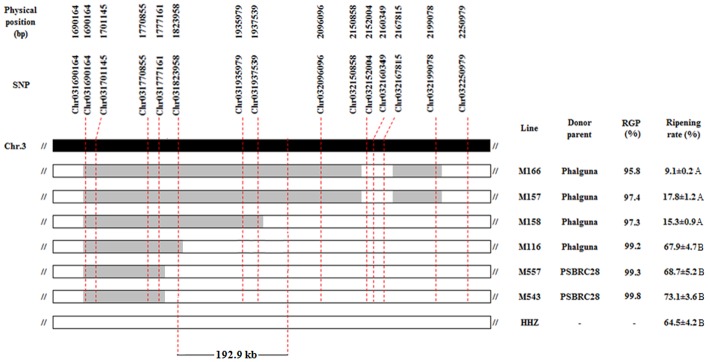
The physical location of *qCT-3-2*. The black colored strip represents chromosome 3 while the grey and white strips indicated the homozygous genotypes of the donor and recurrent parent, respectively. Allele frequency indicated percentage of the alleles derived from HHZ comparing with the entire genome. The phenotypic differences between seven lines were statistically tested using the multiple t-test with Bonferroni adjustment.

Thirteen ILs were first selected out from the IB population based on 41,754 SNPs. The selected ILs had overlapping introgressed segments and covered the genomic region of *qCT-3-2*. Then, we screened again the raw sequencing data and obtained 0.4 M SNPs with MAF>0.03 and miss rate <10%. Using these 0.4 M SNPs, we analyzed these 13 selected ILs. The results showed that the RGP varied from 66.8% to 99.2%. Five ILs had RPG higher than 95.0% and were retained as NILs (Fig 6). Multiple t-test revealed that the five NILs showed highly significant difference in SF. M166 and M157 with the Phalguna allele had significantly lower SF than HHZ and the other NILs (Fig 6). M116, M557 and M543 had slightly higher SF than HHZ. Therefore, the *qCT-3-2* was in the region of 192.6 kb, flanked by chr031823958 and chr32096096 on the reference genome of the *japonica* rice variety Nipponbare (Fig 6).

## Discussion

### The characteristics and advantages of the IB population

Our IB population is similar to NAM population in the sense that the component families shared a common parent. The size of the component families varied greatly in MC populations developed. The first maize NAM population had 25 families with 200 lines per family [10]. The first NAM population developed for barley has RILs derived from BC_1_ families of 25 donors and a single recurrent parent. The number of lines in each family varied from 22 to 75 with the mean of 56.8 [24]. Our IB population had eight families and the number of lines varied from 42 to 67 with an average of 58.5 lines per family. However, different from NAM and other multi-cross populations, lines of our IB population were the ones selected for one or more traits. Differences in selection intensity applied and the genetic complexities of selected traits may result in complex population structure and genetic relationships between lines that may have negative effects on association mapping. For instance, the eight component families were found to have significantly different RGPs. The families derived from the donor parents OM1723 and Teqing had significantly lower RGP than other donors. For all the families, RGPs were also different from the expected theoretical value 0.25 of a random population of RILs derived from a BC_1_ population (S1 Table). A significant deviation (excess or deficiency) of the donor allele frequency at single locus from the expected level implies a positive selection favoring the donor allele (in excess), or negative selection against the donor allele (in deficiency) [35]. When all 479 ILs derived from the eight donors in the same genetic background (HHZ) were pooled as an IB population, the introgressed segments covered almost the whole genome although uneven distribution across genome was seen. For instance, the RGP of chromosome 1 was the lowest while that of chromosome 11 was highest (S2 Table). Obviously, the target trait-specific ILs resulting from two rounds of selection against drought, salinity or submergence tolerances could not be regarded as a approximately random backcross inbred line population, even for the non-target but potentially correlated traits such as CT. Nevertheless, as demonstrated in the present study, association analysis still can be applied to such breeding populations effectively for identifying QTLs that are directly usable in breeding. In fact, under cold stress, the phenotypic variation showed skewed but continuous distribution (Fig 3). The higher genetic diversity between donors and recipient provides more allelic variation across the whole genome, thus conducive to perform GWAS as suggested by Maurer et al. [24]. What need alludes is, based on the selective introgression breeding strategies [35], the IB lines could be also used to pyramid genes for different abiotic stresses such as tolerances to drought, salinity, and submergence after two rounds of selection for the above traits in developing potential breeding lines either by designed QTL pyramiding or by molecular recurrent selection scheme [44].

### QTLs identified

In this study, six independent QTLs for CT were identified on chromosomes 3, 4 and 12. Except for *qCT-4*, which was associated with only a single SNP, Chr045026663, others were located in the intervals of 88 kb to 600.9 kb (Table 4). Comparing QTLs identified in this study with the previously reported CT-related genes using the *O*. *sativa subsp*. *japonica* Kato GRAMENE annotation sequence map 2009 [45] suggested that the six QTL regions were overlapped or adjacent to 11 CT QTLs reported previously (S4 Fig). For instance, the interval of *qCT-3-1*(~655 kb) harbored gene *Os03g0103300*, which had been cloned and showed positive effect on rice seed germination under low temperature environment [46]. *qCT-3-2* identified in all the four environments in the present study partially overlapped with *qPSST-3* for CT at booting stage under cold-water treatment identified by Suh et al. [8] from the breeding line IR66160-121-4-4-2. The *qCT-3-3* located in the short arm of chromosome 3 is closely adjacent to the CT QTL identified at seedling stage by Zhang et al. [47]. The *qCT-4* was in the region adjacent to the *qCT4-1*, a QTL contributing to CT at the booting stage [7, 48], and *qCTS-4*, a QTL contributing to CT at the seedling stage [49]. The *qCT-12-1* was in the region flanked by markers RM3747 and RM6292, where *qLTG-12* had been reported for CT at germination and seedling stages [47, 50]. The *qCT-12-2* on chromosome 12 was mapped to the region containing *qCTS-12* identified previously for CT at seedling stage [51–53]. CT QTLs, which could be simultaneously detected in different stages under diverse genetic backgrounds, were likely to be more stable and have higher value of application in molecular breeding for CT [2, 12]. For QTL *qCT-3-2*, the favorable allele for CT was from the recurrent parent HHZ that has moderate CT. This result provided further support to the clam that even though modern varieties do not show strong stress tolerances, they do harbor some excellent genes for drought tolerance [14, 54], salt tolerance [55–56], and cold tolerance [51, 57]. Such favorable alleles were found to be ‘hidden’ in the primary gene pool of the rice germplasm, and advanced backcross is an effective way to mine them [31, 58]. It is possible to improve the CT of currently adapted elite varieties by identifying such ‘hidden’ favorable alleles from other elite varieties by introgressing and further pyramiding favorable genes of recurrent and donor parents with molecular marker technologies.

In the present study, *qCT-3-2* was repeatedly identified under four natural low temperature environments, suggesting it was a stable QTL for CT at the booting stage. The *qCT-3-2* was fine mapped to a credible interval of 192.9 kb using high-density SNPs and NILs (Fig 6). In order to fully understand the expression features of *qCT-3-2* at different developmental stages, the five NILs selected in this study are being precisely phenotyped under controlled low temperature at germination and seedling stages. Mapping population has been developed using the NILs for further narrowing down the *qCT-3-2* region and eventually cloning the responsible gene.

## Conclusion

We reported here the use of an IB population consisting of selected ILs derived from eight BC families with the same recurrent parent in identifying and fine-mapping QTLs for CT at booting stage. We could successfully identify six QTLs. The stably expressed QTL, *qCT-3-2*, was fine mapped and narrowed down to approximate 192.9 kb on the reference genome. These QTLs, particularly *qCT-3-2*, are important for developing varieties with CT at booting stage, which are highly demanded by rice production especially in the temperate and high altitude regions.

## Supporting Information

S1 Fig Graphical presentation of genotypes of the IB population based on 41,754 SNPs. This figure is divided into 8 areas associating with 8 families from the top down. Blue indicates the recipient genotype HHZ, other colors indicate segments from different donors and yellow indicates heterozygous genotype.
(TIF)

S2 Fig Genetic similarity amongst ILs and families based on 41,754 SNPs. (TIF)

S3 Fig Genome-wide average LD decay of the HHZ IB population. Genome-wide LD decay rates were estimated at ~400 kb, where the *r* ^*2*^ was 0.50.
(TIF)

S4 Fig Comparison of QTLs for CT at booting stage identified in the HHZ IB population with the previous reported ones. (TIF)

S1 Table The recurrent genome percentage among eight families. (DOCX)

S2 Table The recurrent genome percentage among 12 chromosomes. (DOCX)

S3 Table Phenotypic correlations between four testing environments. (DOCX)
