# Supplementary figures and images for: Identification and Fine Mapping of a Stably Expressed QTL for Cold Tolerance at the Booting Stage Using an Interconnected Breeding Population in Rice
Source: PLoS One. 2015 Dec 29;10(12):e0145704. doi: 10.1371/journal.pone.0145704 (PMC4703131; doi:10.1371/journal.pone.0145704)

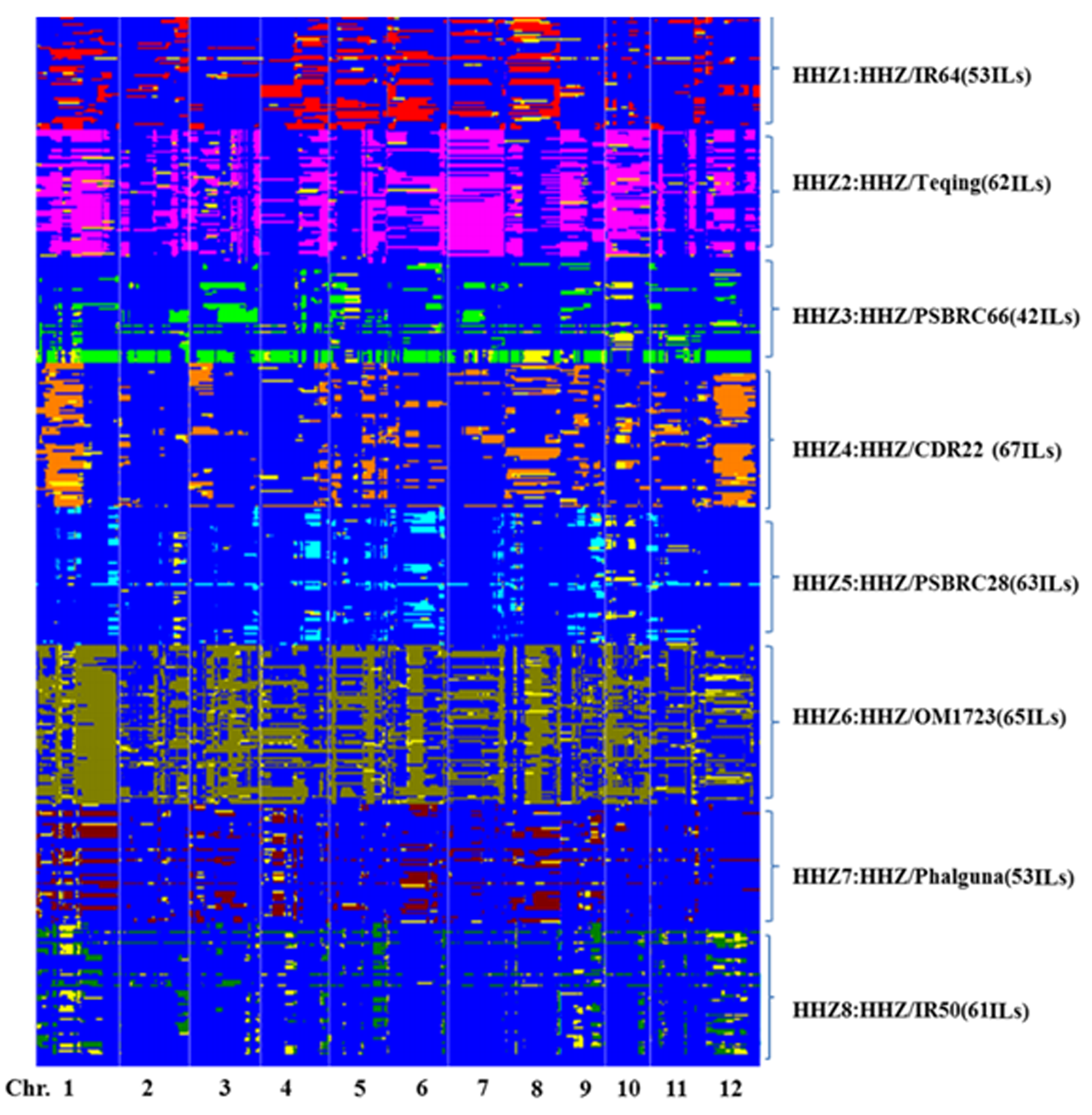

Supplement: S1 Fig — This figure is divided into 8 areas associating with 8 families from the top down. Blue indicates the recipient genotype HHZ, other colors indicate segments from different donors and yellow indicates heterozygous genotype. (TIF) [file pone.0145704.s001.tif]

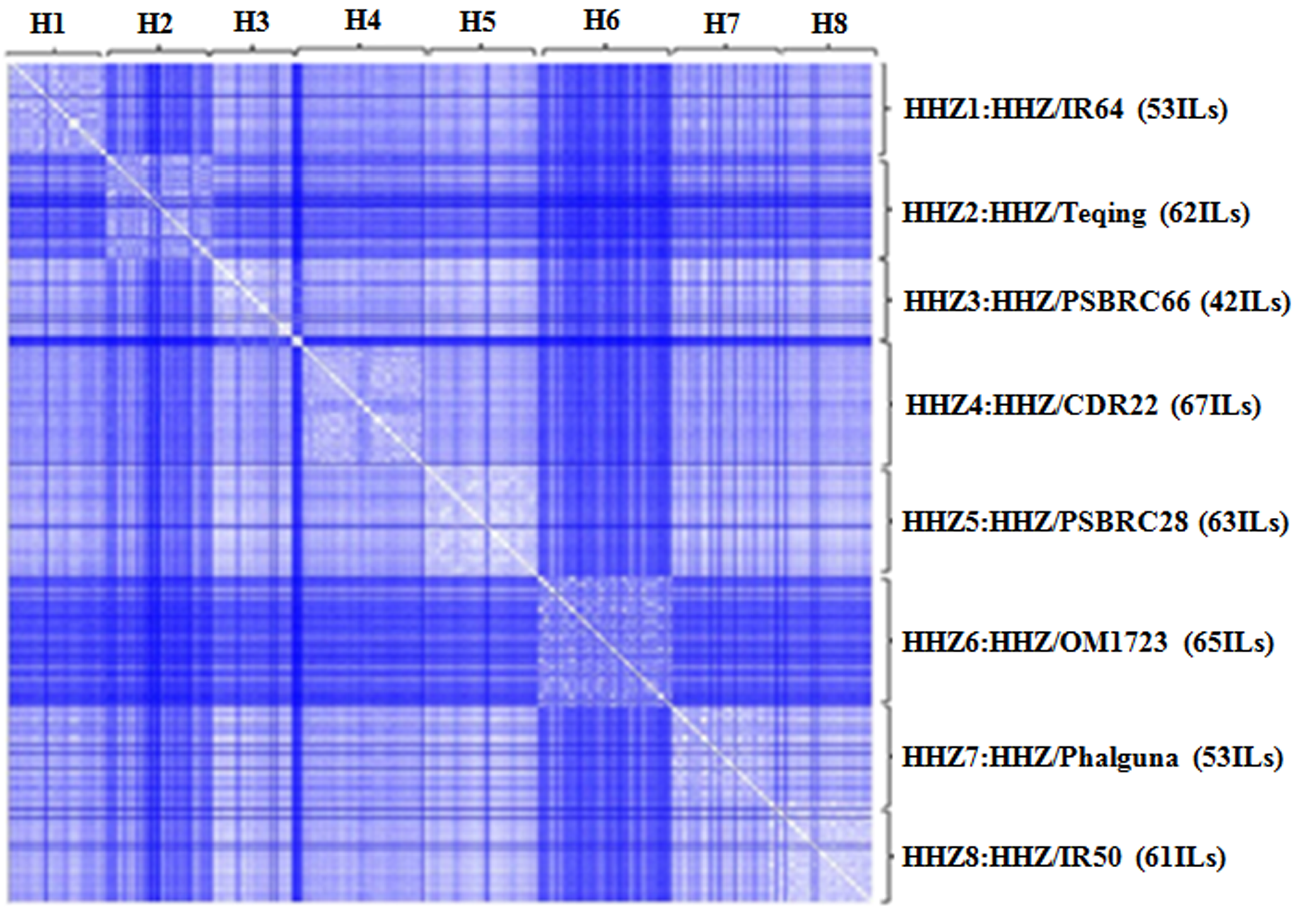

Supplement: S2 Fig — (TIF) [file pone.0145704.s002.tif]

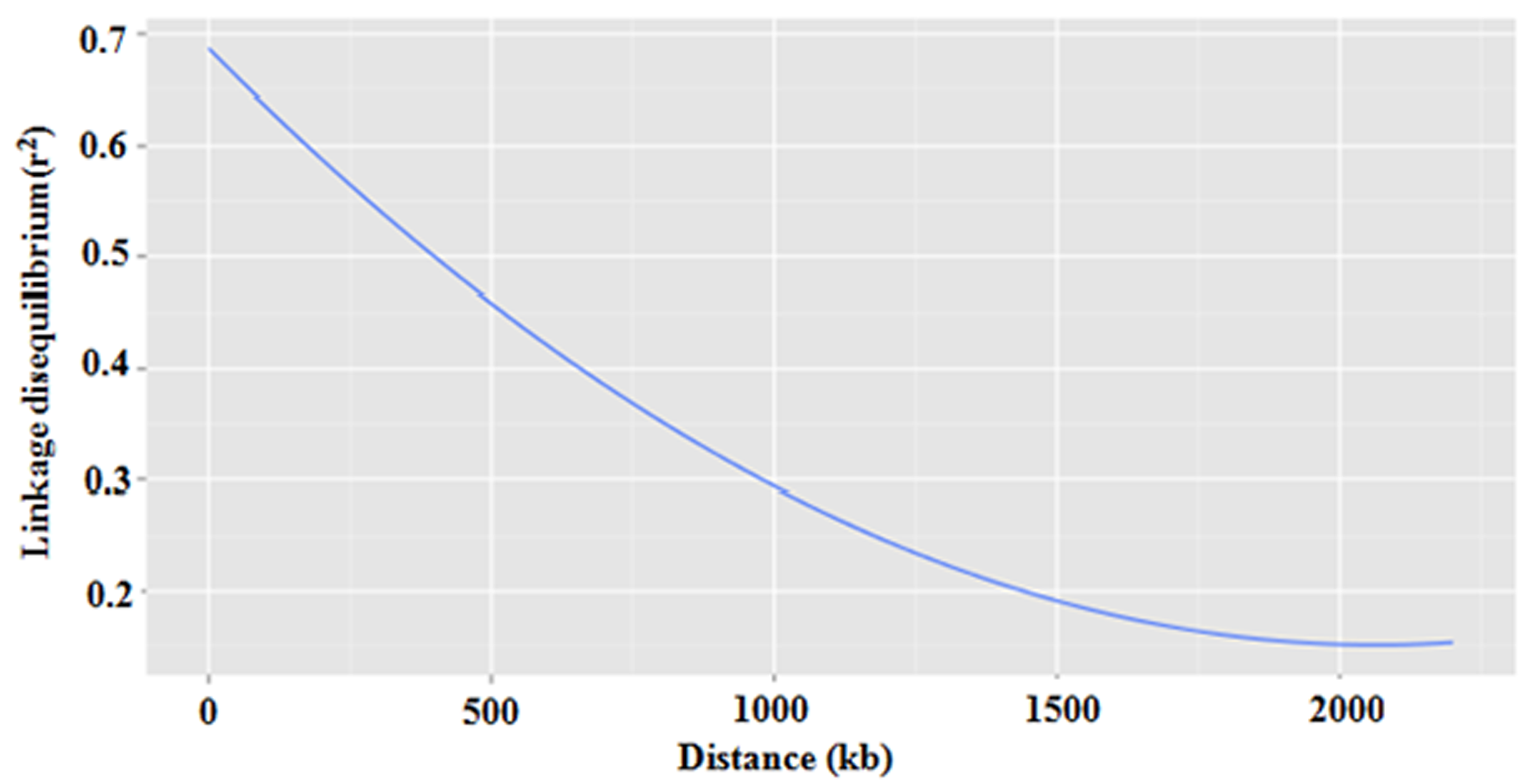

Supplement: S3 Fig — Genome-wide LD decay rates were estimated at ~400 kb, where the r 2 was 0.50. (TIF) [file pone.0145704.s003.tif]

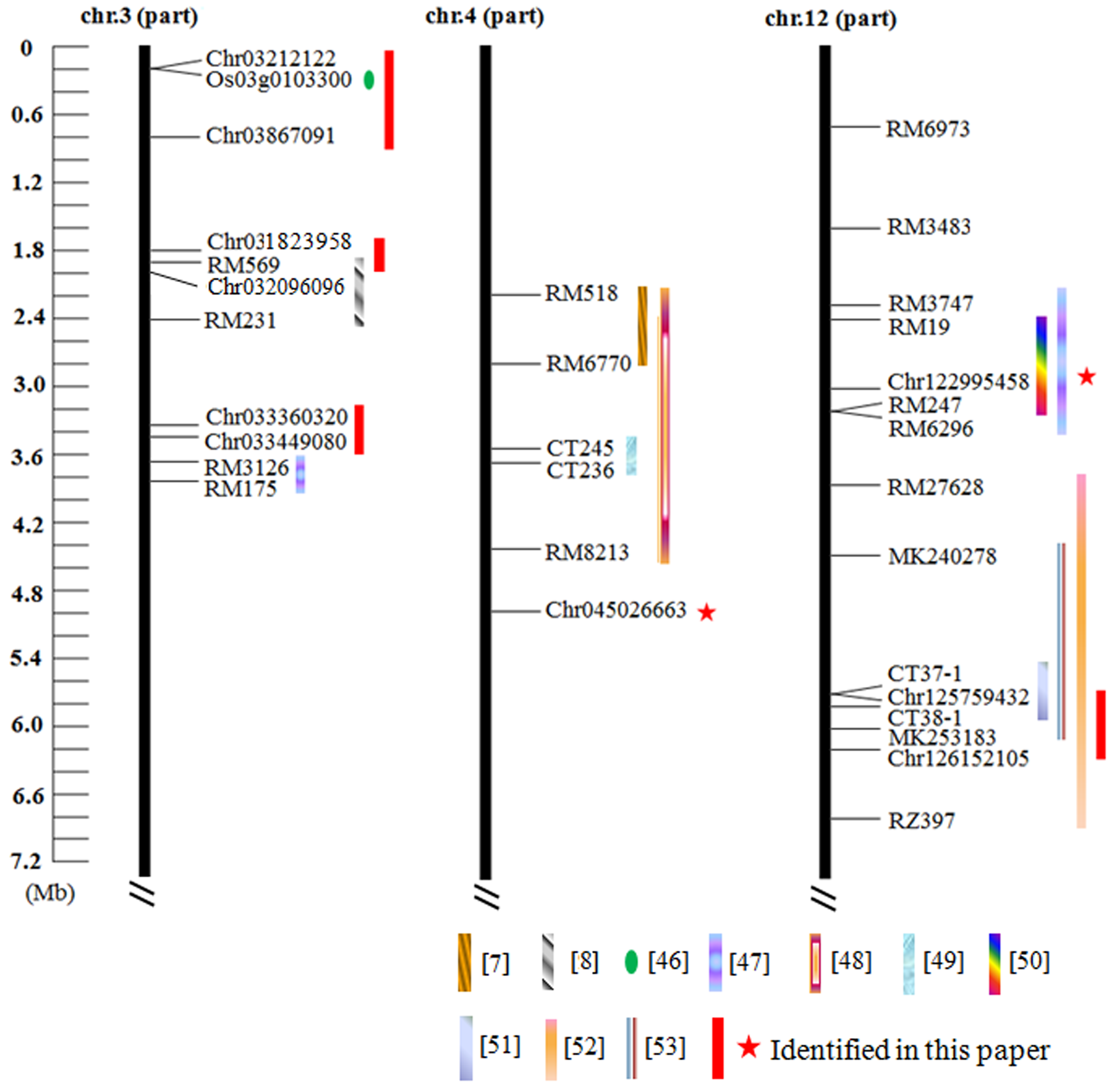

Supplement: S4 Fig — (TIF) [file pone.0145704.s004.tif]
