# Supplementary material for: Identification and Fine Mapping of a Stably Expressed QTL for Cold Tolerance at the Booting Stage Using an Interconnected Breeding Population in Rice
Source: PLoS One. 2015 Dec 29;10(12):e0145704. doi: 10.1371/journal.pone.0145704 (PMC4703131; doi:10.1371/journal.pone.0145704)
Supplement: S1 Table — (DOCX) [file pone.0145704.s005.docx]

**S1 Table. The recurrent genome percentage among families.**

| Family | Cross | Mean (%) | DMRT Grouping |
| --- | --- | --- | --- |
| H6 | HHZ/OM1723 | 55.7 | A |
| H2 | HHZ/Teqing | 62.9 | B |
| eightH1 | HHZ/IR64 | 78.9 | C |
| H3 | HHZ/PSBRC66 | 80.4 | D |
| H4 | HHZ/CDR22 | 80.5 | D |
| H7 | HHZ/Phalguna | 84.4 | E |
| H5 | HHZ/PSBRC28 | 89.2 | F |
| H8 | HHZ /IR50 | 89.7 | F |

Multiple comparisons were conducted using Duncan’s multiple range test (DMRT), where the same letter indicates non-significant differences while different letters mean significant differences at P<=0.05 level.
