# Supplementary material for: Identification and Fine Mapping of a Stably Expressed QTL for Cold Tolerance at the Booting Stage Using an Interconnected Breeding Population in Rice
Source: PLoS One. 2015 Dec 29;10(12):e0145704. doi: 10.1371/journal.pone.0145704 (PMC4703131; doi:10.1371/journal.pone.0145704)
Supplement: S2 Table — (DOCX) [file pone.0145704.s006.docx]

**S2 Table. The recurrent genome percentage among 12 chromosomes.**

| Chromosome | Mean (%) | Duncan Grouping |
| --- | --- | --- |
| 1 | 68.4 | A |
| 6 | 74.9 | B |
| 8 | 75.0 | B |
| 12 | 76.3 | C |
| 10 | 76.8 | C |
| 5 | 77.1 | C |
| 4 | 78.3 | D |
| 3 | 78.5 | D |
| 9 | 78.6 | D |
| 7 | 78.9 | D |
| 2 | 84.6 | E |
| 11 | 85.5 | E |

Multiple comparisons were conducted using Duncan’s multiple range test (DMRT), where the same letter indicates non-significant differences while different letters mean significant differences at P<=0.05 level.
