# Supplementary material for: Identification and Fine Mapping of a Stably Expressed QTL for Cold Tolerance at the Booting Stage Using an Interconnected Breeding Population in Rice
Source: PLoS One. 2015 Dec 29;10(12):e0145704. doi: 10.1371/journal.pone.0145704 (PMC4703131; doi:10.1371/journal.pone.0145704)
Supplement: S3 Table — (DOCX) [file pone.0145704.s007.docx]

**S3 Table. Phenotypic correlations between four testing environments.**

| Environment | SM2014 | XD2014 | YX2013 |
| --- | --- | --- | --- |
| XD2014 | 0.634*** |  |  |
| YX2013 | 0.597*** | 0.587*** |  |
| XD2013 | 0.634*** | 0.59*** | 0.586*** |
